# *UBQLN4* Is a Prognostic Biomarker in Intrahepatic Cholangiocarcinoma That Is Associated with Cisplatin Resistance

**DOI:** 10.3390/cells15181697

**Published:** 2026-09-18

**Authors:** Kodai Abe, Kelly Chong, Yuta Abe, Minoru Kitago, Motonori Edanami, Yosuke Uematsu, Yohei Masugi, Akihisa Ueno, Matias A. Bustos, Anton J. Bilchik, Yosef Shiloh, Yuko Kitagawa, Dave S. B. Hoon

**Affiliations:** 1 Department of Translational Molecular Medicine, Saint John’s Cancer Institute (SJCI), Saint John’s Health Center (SJHC), Providence Health System (PHS), Santa Monica, CA 90404, USA; kelly.chong@providence.org (K.C.); matias.bustos@providence.org (M.A.B.); dave.hoon@lundquist.org (D.S.B.H.); 2 Department of Surgery, Keio University School of Medicine, Shinjuku 160-8582, Tokyo, Japan; abey3666@gmail.com (Y.A.); dragonpegasus427@gmail.com (M.K.); mamemamenorinori@gmail.com (M.E.); u.yosuke9872@gmail.com (Y.U.); kitagawa.a3@keio.jp (Y.K.); 3 Department of Pathology, Keio University School of Medicine, Shinjuku 160-8582, Tokyo, Japan; masugi.z6@gmail.com; 4 Department of Pathology, Tokai University School of Medicine, Isehara 259-1193, Kanagawa, Japan; akihisaueno@gmail.com; 5 Division of Diagnostic Pathology, Keio University School of Medicine, Shinjuku 160-8582, Tokyo, Japan; 6 Department of Translational and Precision Medicine, Saint John’s Cancer Institute (SJCI), Saint John’s Health Center (SJHC), Providence Health System (PHS), Santa Monica, CA 90404, USA; 7 Department of Gastrointestinal and Hepatobiliary Surgery, Saint John’s Cancer Institute (SJCI), Saint John’s Health Center (SJHC), Providence Health System (PHS), Santa Monica, CA 90404, USA; anton.bilchik@providence.org; 8 David and Inez Myers Laboratory for Cancer Genetics, Department of Human Genetics and Computational Medicine, Gray Faculty of Medical & Health Sciences, Tel Aviv University, Tel Aviv 69978, Israel; yossih@tauex.tau.ac.il; 9 Advanced Therapeutics and Biomedical Technologies, The Lundquist Institute of Biomedical Innovation, Harbor-UCLA Medical Center, Torrance, CA 90504, USA

**Keywords:** cisplatin, CNV, intrahepatic cholangiocarcinoma, neoadjuvant chemotherapy, UBQLN4

## Abstract

**Highlights:**

**What are the main findings?**

- *Ubiquilin-4* (*UBQLN4*) mRNA expression was significantly elevated in intrahepatic cholangiocarcinoma (iCCA) compared to normal liver tissue.
- Elevated *UBQLN4* mRNA expression was associated with shorter recurrence-free and overall survival across independent multicenter iCCA patient cohorts.

**What are the implications of the main findings?**

- These findings identify UBQLN4 as a clinically relevant biomarker associated with aggressive tumor progression and cisplatin resistance in iCCA.
- Assessment of *UBQLN4* mRNA expression in tumor tissues may improve risk stratification after curative-intent resection and help identify iCCA patients who may benefit from intensified surveillance or future biomarker-guided therapeutic strategies.

**Abstract:**

Background: Intrahepatic cholangiocarcinoma (iCCA) is an aggressive biliary tract malignancy with limited treatment options and poor prognosis. Platinum-based drugs are one of the major therapies; however, resistance remains a major therapeutic challenge. Ubiquilin-4 (UBQLN4), a ubiquitin-binding protein implicated in cancer progression and DNA damage responses, is located at chromosome 1q22, a region recurrently amplified in several cancers. We investigated the clinical and functional relevance of UBQLN4 in iCCA. Methods: Public datasets (TCGA-CHOL and GSE107943) were analyzed to identify genomic alterations and *UBQLN4* expression in iCCA. *UBQLN4* mRNA expression was evaluated in an independent clinical cohort and correlated with patient outcomes. Functional studies using iCCA cell lines and three-dimensional spheroid models examined the role of UBQLN4 in cisplatin resistance. Results: Chromosome 1q22, containing *UBQLN4*, was among the recurrently amplified regions in TCGA-CHOL. *UBQLN4* mRNA expression correlated with copy number amplification and was significantly higher in iCCA than in normal liver tissue in both datasets. In the validation cohort, higher *UBQLN4* mRNA expression was associated with shorter recurrence-free survival (*p* = 0.025) and remained associated with shorter recurrence-free survival after adjustment for clinicopathological factors (HR = 2.44; 95% CI 1.14–5.20; *p* = 0.022). Higher UBQLN4 expression was also associated with poorer pathological response to cisplatin-containing neoadjuvant chemotherapy. In iCCA cell lines, *UBQLN4* knockdown increased cisplatin sensitivity, whereas cisplatin-resistant derivatives showed increased UBQLN4 expression. Conclusions: Elevated *UBQLN4* expression is associated with adverse clinical outcomes and reduced response to cisplatin-containing chemotherapy in iCCA, while functional studies support a potential role for UBQLN4 in modulating cisplatin sensitivity. These findings identify UBQLN4 as a potential biomarker and therapeutic candidate that warrants further independent and prospective validation.

## 1. Introduction

Biliary tract cancers (BTCs) remain highly lethal malignancies that are frequently diagnosed at advanced stages and with aggressive histopathological features [1,2]. Intrahepatic cholangiocarcinoma (iCCA), a major subtype of BTCs, is particularly challenging because it often lacks early clinical symptoms and reliable biomarkers for early detection, resulting in delayed diagnosis and poor clinical outcomes [2]. Even among patients who undergo potentially curative surgical resection, recurrence is common, and long-term survival remains limited, underscoring the aggressive biological behavior of iCCA and the substantial unmet clinical need for improved disease detection and management [3]. Moreover, the global incidence of iCCA has increased steadily in recent decades particularly in Asia and Europe, with high rates of postoperative recurrence and disease-related mortality, despite recent advances in systemic therapies [4,5].

Several molecular alterations have been identified in iCCA, including fibroblast growth factor receptor 2 (FGFR2) fusions or rearrangements (<10%, [6]) and isocitrate dehydrogenase 1 (IDH1) mutations (~13%, [7]). These genomic alterations occur at relatively low frequencies and are typically targetable only after disease progression on standard of care treatments including, DNA-damage orientated therapies (cisplatin- and gemcitabine-based chemotherapies) or immune checkpoint inhibitor immunotherapies [8,9]. Furthermore, the clinical efficacy of these targeted agents remains limited, largely due to pronounced tumor heterogeneity and the rapid onset of chemoresistance during treatment [10]. Consequently, there is a critical unmet need to identify robust biomarkers that can stratify iCCA patients with aggressive disease, predict treatment response, and inform therapeutic decision-making.

Cisplatin, a DNA damage drug, remains a cornerstone of systemic therapy for advanced iCCA; however, intrinsic and acquired resistance to platinum-based chemotherapy represents a major barrier to durable clinical benefit [11]. Cisplatin exerts its cytotoxic effects primarily by inducing DNA crosslinks, thereby activating DNA damage response (DDR) signaling pathways [12]. Dysregulation of DDR components has been increasingly implicated in therapeutic resistance across multiple cancer types, suggesting that altered DNA damage repair capacity may contribute to poor treatment outcomes in iCCA [13].

Previous studies from our groups identified that ubiquilin-4 (*UBQLN4*) was associated with prognosis and chemotherapy response in carcinoma such as triple-negative breast cancer (TNBC), ovarian cancer, and esophageal squamous cell carcinoma (ESCC, [14,15]). *UBQLN4* gene (previously referred to as *CIP75*) is located at the 1q22 chromosome region; the protein binds to ubiquitinated proteins and then facilitates their translocation to the 26S proteasome for degradation [16,17]. A key finding we previously demonstrated was that UBQLN4 is an important factor regulating ubiquitinated protein degradation during DDR [18]. We have also shown that *UBQLN4* gene is amplified and highly expressed across different histological types of solid tumors in The Cancer Genome Atlas (TCGA) datasets [19].

Based on these observations, we hypothesized that elevated UBQLN4 levels are associated with the functional modulation of DDR signaling, thereby influencing iCCA progression and cisplatin resistance. In this multicenter retrospective study, we investigated the clinical utility of UBQLN4 in iCCA patients. Our findings highlighted UBQLN4 as a novel biomarker with potential clinical utility that links genomic amplification to therapeutic resistance, with potential implications for risk stratification and treatment optimization for patients with iCCA.

## 2. Materials and Methods

### 2.1. Public iCCA Datasets

The following public datasets were used for analyses; TCGA-CHOL and GSE107943 [20]. In the TCGA-CHOL cohort, copy number variation (CNV) data and mRNA expression data were obtained and only patients with iCCA were included in downstream analysis, while the other cholangiocarcinoma histological subtypes such as gallbladder carcinoma and extrahepatic cholangiocarcinoma were excluded from further analysis to minimize potential heterogeneity arising from their distinct anatomical and molecular characteristics. CNV analysis was performed by Genomic Identification of Significant Targets in Cancer (GISTIC) 2.0 analysis (Broad Institute, Cambridge, MA, USA) [21]. All data of TCGA-CHOL were acquired and extracted from UCSC Xena (https://xenabrowser.net/datapages/ (accessed on 24 April 2024)). The GSE107943 dataset was used exclusively for mRNA expression analyses and was not included in clinicopathological summary tables or survival analyses.

### 2.2. Gene Set Enrichment Analysis (GSEA)

Public iCCA datasets with RNA-seq (TCGA-CHOL and GSE107943) data available were evaluated for *UBQLN4* expression. For all datasets, cholangiocarcinoma tumor samples were split into *UBQLN4*-high and *UBQLN4*-low groups using median *UBQLN4* expression, and differential gene expression analysis was performed. For RNA-seq datasets, samples were split using median DESeq2-normalized expression values, followed by differential expression analysis using DESeq2 (1.50.2). For the RNA microarray datasets, samples were split by median normalized expression values, followed by differential expression analysis using limma (edgeR 3.40.2). Volcano plots were generated using ggplot2 (3.5.2) and EnhancedVolcano (1.20.0). GSEA was performed using the fgsea R package (1.28.0), where genes were ranked using the signed negative log10 *p*-value (RANK = sign(log2FC) × −log10(*p*-value)) [22]. Hallmark gene sets from MSigDB [23] were used for the GSEA.

### 2.3. Patients and Specimens

This retrospective study enrolled iCCA patients (*n* = 66) who underwent surgery between 2011 and 2025 at SJCI, Santa Monica, CA, USA, and Keio University Hospital, Tokyo, Japan. The clinical background of iCCA patients is shown in Table S1, whereby formalin-fixed paraffin-embedded (FFPE) tissues were assessed from all patients. This study was approved by Western IRB: HOOD-PEMEL-0203 (IRB 20072153), MORD-RTPCR-0995, and Keio University Hospital’s IRB (No. 20241017), according to the Declaration of Helsinki. Patients who were diagnosed as combined iCCA and hepatocellular carcinoma, iCCA arisen from intraductal papillary neoplasm of bile duct (IPNB), and cholangiolocellular carcinoma (CoCC) were excluded from the analysis. Among the 66 patients, 16 patients with locally advanced iCCA and radiologically suspected lymph node metastases received neoadjuvant chemotherapy (NAC) before surgical resection. All 16 patients were classified as clinical stage III according to the American Joint Committee on Cancer (AJCC) 8th edition staging system. NAC consisted of gemcitabine (1000 mg/m^2^) and cisplatin (25 mg/m^2^), administered on days 1 and 8 of each 3-week cycle for a total of two cycles before surgical resection. This cohort was retrospectively analyzed to explore the association between UBQLN4 expression and pathological response to NAC. Pathological response to NAC was retrospectively assessed in resected specimens according to the Evans grading system [24,25]. Patients with Evans Grade IIb or greater responses (more than 50% of tumor cell destruction) were classified as pathological responders, whereas those with Grade IIa or lower responses were classified as non-responders.

### 2.4. Quantitative Reverse Transcription PCR

RNA was extracted from cell lines using the ZR-Duet DNA/RNA MiniPrep kit (Zymo Research, Tustin, CA, USA) and from FFPE tissue specimens using the Maxwell RSC RNA FFPE Kit (Promega, Madison, WI, USA). Turbo DNase was used to remove DNA contamination. cDNA synthesis was conducted by M-MLV reverse transcriptase (Promega, Madison, WI, USA) with Oligo (dt) primer and random primers. The PCR was performed using PerfecTa SYBR Green FastMix (VWR International, Randor, PA, USA) in LightCycler 96 System (Roche, Mannheim, Germany). The sequences for all primers utilized are listed in Table S2. Quantitative expression was performed using human succinate dehydrogenase complex, subunit A as a reference gene and for 2^(−ΔΔCt)^ normalization [26].

### 2.5. Cell Lines

An RBE cell line (Cytion, Sioux Falls, SD, USA, human female adenocarcinoma-derived iCCA) was cultured in RPMI (Sigma Aldrich, St. Louis, MO, USA) and supplemented with 10% fetal bovine serum (FBS), 1% penicillin–streptomycin, and fungizone. An HCCC9810 cell line (Amsbio LLC, Cambridge, MA, USA, human female epithelial-like iCCA) was cultured in RPMI 1640 and supplemented with 10% FBS and 1% penicillin–streptomycin. All experiments were performed with mycoplasma-free cell lines [27]. To establish the cisplatin-resistant (CR) iCCA cell lines, RBE and HCCC9810 cell lines were cultured at 1.0 μM for cisplatin (Med Chem Express, Monmouth Junction, NJ, USA) as initial concentration for 72 h. Then, they were changed to the normal medium and the cell lines were cultured until the resistant clones proliferated at about 80% confluence. When the cells reached 80–90% confluence, the cells were washed with PBS, passed and seeded (0.5 ×10^6^ cells). After 24 h incubation, the cells were re-treated at higher dose concentrations of cisplatin. The cycle was repeated for the following doses, until resistant cell lines grew in 5.0 μM for cisplatin. Finally, we continued to incubate RBE and HCCC9810 at 1.0 μM for cisplatin for more than 4 wks (up to 10 wks).

### 2.6. Cell Viability Assay

RBE and HCCC9810 cells were seeded for 2.5 × 10^3^ cells per well in 96-well culture plates (Thermo Fisher Scientific, Waltham, MA, USA). Both cell lines were treated with different concentrations of cisplatin. The number of viable cells was assessed using a Cell Titer-Glo luminescent cell viability assay (Promega) according to the manufacturer’s instructions as previously described [28]. Dose–response data were normalized to the mean viability of untreated control cells (100%). Half-maximal inhibitory concentration (IC50) values were estimated by nonlinear regression using a log-logistic dose–response model with the lower and upper asymptotes fixed at 0% and 100%, respectively, using the drc package in R. The resistance index was calculated as the ratio of the IC50 of the cisplatin-resistant derivative to that of the corresponding parental cell line.

### 2.7. Three-Dimensional Spheroid Assay

RBE and HCCC9810 cells (5.0 × 10^3^ per well) were suspended in 100 μL of medium and cultured using a three-dimensional (3D) spheroid 96-well microplate (Corning Inc., Corning, NY, USA) as described previously [28]. The medium was replaced on day 2 and then every two days. On day 5, cisplatin was administered in each well.

### 2.8. Small Interfering RNA

RBE and HCCC9810 (2.5 × 10^5^ cells per well) were transfected with 25 nM ON-TARGET plus SMART pool siRNA (Dharmacon, Lafayette, CO, USA, #L-017599-00-0005) to downregulate human *UBQLN4* (Dharmacon, #L-021178-01-0005) or non-targeting pool siRNA as a control using jet-PRIME transfection reagent (Polyplus-transfection, Illkirch, France).

### 2.9. Western Blot

A conventional Western blot (WB) experiment was performed as previously described [26]. The proteins were extracted with lysis buffer (150 mM NaCl, 100 mM Tris/HCl pH 8, 1% NP-40, protease inhibitors, and phosphatase inhibitors), and the protein concentration was calculated using a bicinchoninic acid assay. The results were analyzed and quantified using the ImageJ.JS 1.53m (web-based version of ImageJ; ImJoy Team). The primary Abs are listed in Table S3. All uncropped WB images are shown in Figure S2.

### 2.10. Multiplex Immunohistochemistry and Quantitative Analysis

FFPE tissue sections (5 μm) were deparaffinized in xylene and then rehydrated in 100, 100, and 80% ethanol as previously described [28]. Briefly, the sections were pre-incubated using the Abs described in Table S2. The antigenic binding sites were visualized using the Opal dye. Opal-520 and Opal-690 (Akoya Biosciences, Brooklyn, NY, USA) were incubated with a specific primary Ab as described in Table S3. In each slide, average six photographs per sample were captured at 40× magnification. Data was analyzed using the image software Mantra 1.0.5 (Perkin Elmer, Waltham, MA, USA) and inForm software 2.6.0 (Akoya Biosciences)-based active machine learning algorithm with a pre-visual cutoff, followed by single-cell-based mean pixel fluorescence intensity to achieve accuracy [28].

### 2.11. Biostatistical Analysis

Statistical analyses were performed using R version 4.4.0. Comparisons between two independent groups were performed using Student’s *t*-test, Welch’s *t*-test, or the Wilcoxon rank-sum test, as appropriate. Welch’s *t*-test was used when equal variances could not be assumed, whereas the Wilcoxon rank-sum test was used for nonparametric comparisons. Two-way analysis of variance (ANOVA) was used for experiments involving two independent factors. Pearson’s correlation coefficient was used to assess linear associations between continuous variables.

For survival analyses, patients were divided into high- and low-expression groups according to the median *UBQLN4* mRNA expression level. Overall survival (OS) and recurrence-free survival (RFS) were estimated using the Kaplan–Meier method and compared using the log-rank test. Cox proportional hazards regression models were used to estimate hazard ratios (HRs) and 95% confidence intervals (CIs) in univariable and multivariable analyses. As a sensitivity analysis, *UBQLN4* mRNA expression was additionally analyzed as a continuous variable standardized to one standard deviation. All statistical tests were two-sided, and *p* < 0.05 was considered statistically significant.

## 3. Results

### 3.1. UBQLN4 Was Highly Amplified and Upregulated in iCCA Tissues

**Figure 1:**
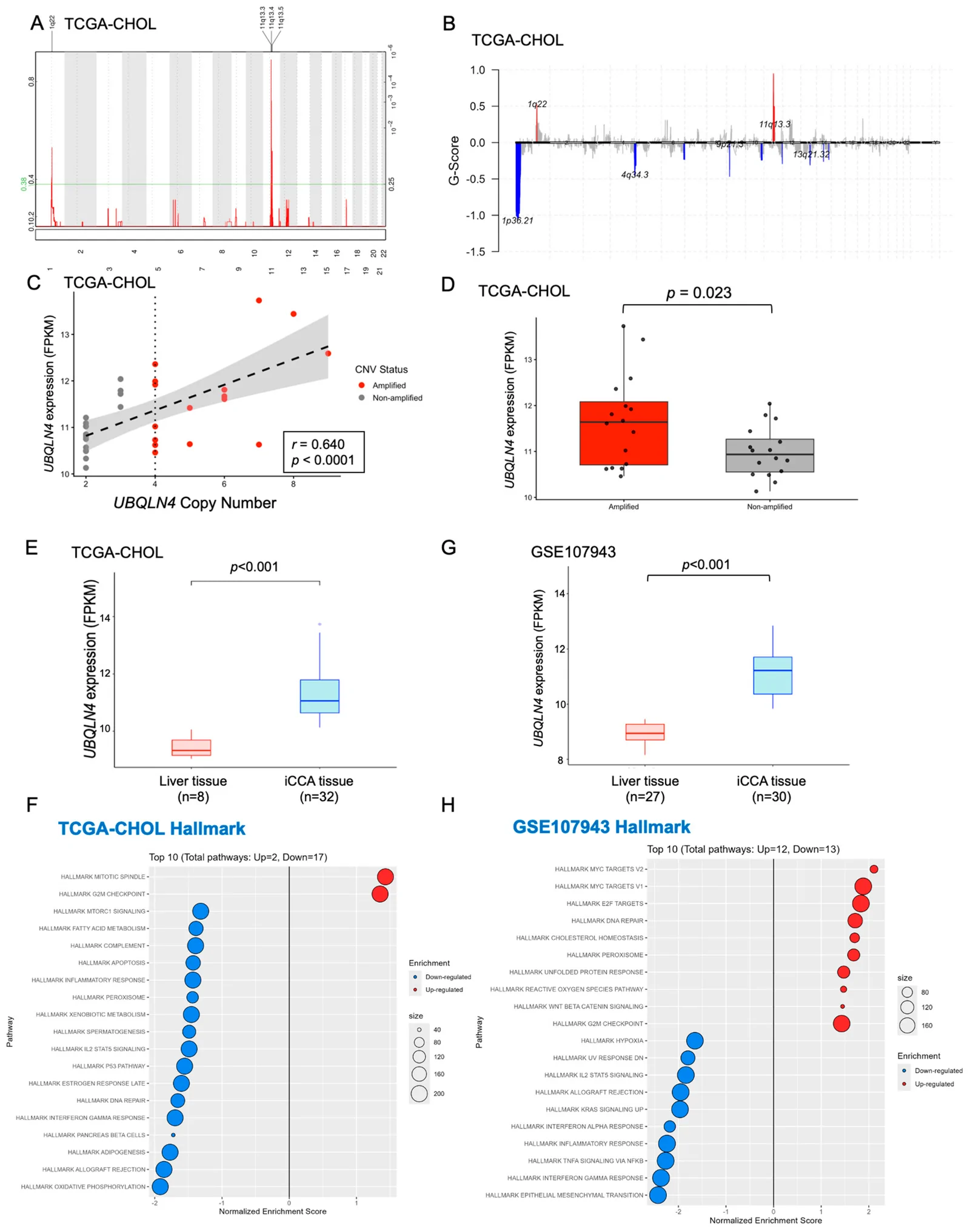
**Genomic amplification and upregulation of *UBQLN4* in iCCA.** (**A**,**B**) GISTIC2.0 analysis for genome-wide copy number variation (CNV) profile of TCGA-CHOL dataset, and G-score plot. (**C**) Correlation analysis between *UBQLN4* copy number and mRNA expression in TCGA-CHOL dataset. (**D**) Comparison of *UBQLN4* mRNA expression in TCGA-CHOL dataset, stratified by CNV status. (**E**) Comparison of *UBQLN4* expression between iCCA tumor tissues and adjacent normal liver tissues in TCGA-CHOL dataset. (**F**) GSEA Hallmark analysis of TCGA-CHOL (**G**) Validation of *UBQLN4* mRNA expression in GSE107943 dataset. (**H**) GSEA Hallmark analysis of the GSE107943 datasets. Statistical significance was determined using Student’s *t*-test (**D**,**E**,**G**) or Pearson correlation test (**C**). Data are presented as mean ± SD (**D**,**E**,**G**).

GISTIC 2.0 analysis using the TCGA-CHOL dataset showed that 1q22 chromosomal regions were amplified in iCCA tumors (G score = 0.5; *q*-value = 0.039; Figure 1A,B), consistent with previous studies [19]. *UBQLN4*, which is located on the 1q22 chromosomal region, was also amplified (16 of 32, 50%) in iCCA tumor tissues. There was also a significant positive correlation between copy number (CN) and mRNA expression levels of *UBQLN4* in the TCGA-CHOL dataset (r = 0.64; *p* < 0.001; Figure 1C). *UBQLN4* mRNA expression levels were significantly higher in tissues with *UBQLN4* amplification than in tissues without *UBQLN4* amplification (*p* = 0.023; Figure 1D). *UBQLN4* mRNA expression levels were also higher in iCCA tissues than in adjacent normal liver samples in the TCGA-CHOL dataset (*p* < 0.001; Figure 1E). This significance remained evident in the GSE107943 (*p* < 0.001; Figure 1F). To explore the biological pathways associated with *UBQLN4* mRNA expression, we performed Hallmark GSEA in the TCGA-CHOL and GSE107943 cohorts. In both datasets, tumors with high *UBQLN4* mRNA expression demonstrated significant enrichment of cell cycle-related pathways, including G2M checkpoint and mitotic spindle signaling (Figure 1G,H; Table S4A,B). These findings suggested that elevated *UBQLN4* mRNA expression is associated with the activation of proliferative and cell cycle regulatory pathways in iCCA.

### 3.2. Clinical Significance of UBQLN4 mRNA Upregulation in iCCA Tissues

**Figure 2:**
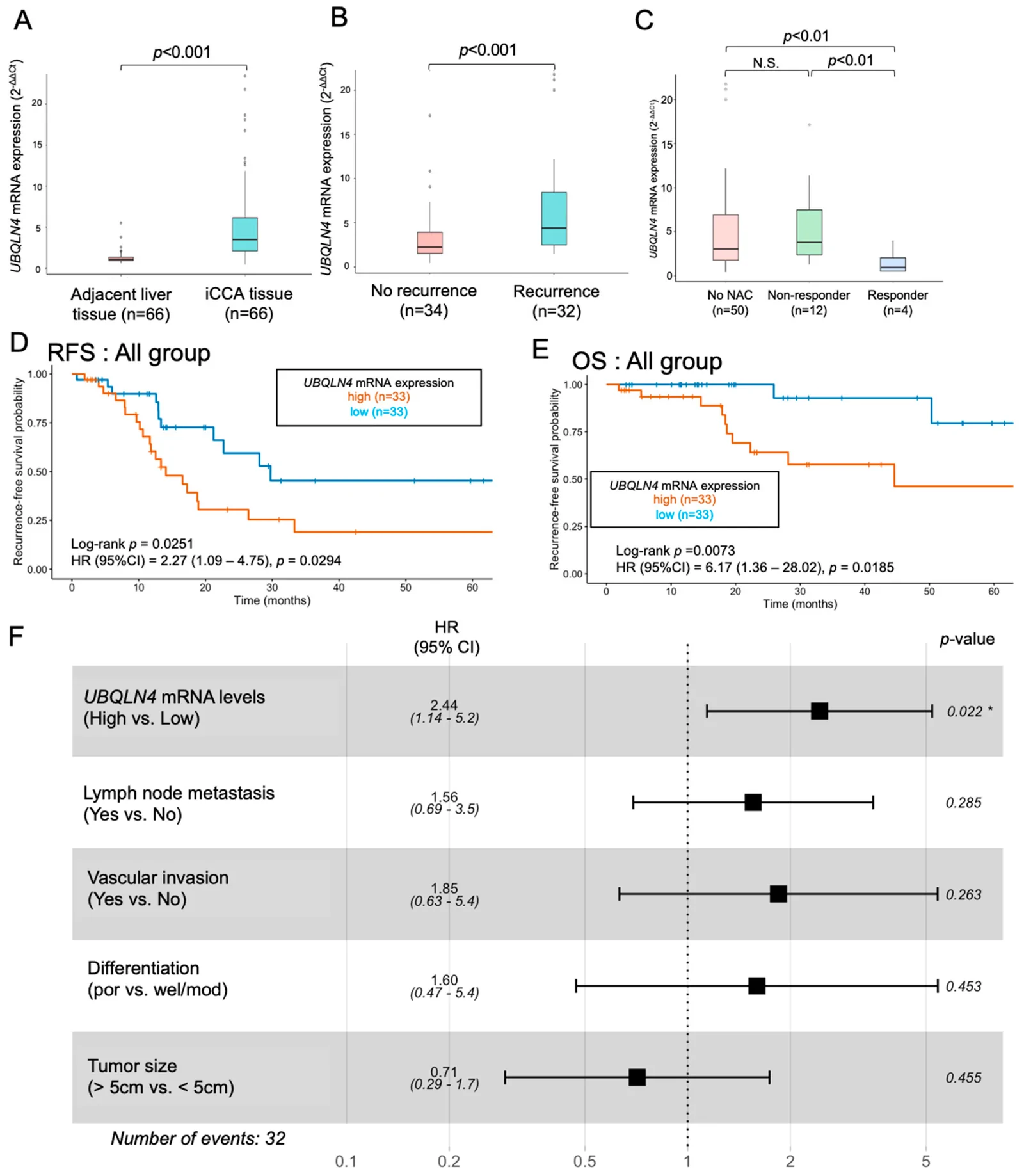
***UBQLN4* mRNA expression is associated with poor prognosis and therapeutic response in iCCA.** (**A**) Comparison of *UBQLN4* mRNA levels in tumor-adjacent normal liver samples and iCCA tissues (*n* = 66) quantified by qRT-PCR. (**B**) Comparison of *UBQLN4* mRNA levels in iCCA patients with and without recurrence. (**C**) *UBQLN4* expression across treatment groups (no NAC, non-responders, and responders). (**D**,**E**) Kaplan–Meier curves for RFS and OS in all iCCA patients’ groups comparing patients with high and low *UBQLN4* levels when considering median values. (**F**) Multivariate cox regression analysis for recurrence in iCCA clinical cohort. The dashed vertical line indicates a hazard ratio of 1.0. Statistical significance was determined using Student’s *t*-test (**A**–**C**) and log-rank test (**D**,**E**). Hazard ratios (HRs) and 95% confidence intervals (CIs) were estimated using multivariable Cox proportional hazards regression analysis, with *p*-values calculated using Wald test (**D**–**F**). Data are presented as mean ± SD (**A**–**C**). N.S., not significant; * *p* < 0.05.

In an independent validation cohort (*n* = 66), *UBQLN4* mRNA levels were found to be significantly elevated in iCCA tissues compared to adjacent normal liver tissues (*p* < 0.001; Figure 2A). Higher *UBQLN4* mRNA expression was observed in patients who experienced postoperative recurrence compared with patients without recurrence (*p* < 0.001; Figure 2B). A subgroup of 16 iCCA patients (16 of 66; 24.2%) received neoadjuvant chemotherapy (NAC; gemcitabine and cisplatin), of which four patients were responders and twelve were non-responders. Despite the limited number of iCCA patients, non-responders had higher *UBQLN4* mRNA expression than responders (Figure 2C). Survival analysis using Kaplan–Meier curves demonstrated that high *UBQLN4* mRNA expression was associated with significantly shorter RFS and OS in the entire cohort (log rank test [*n* = 66]; *p* = 0.025 and *p* = 0.007, respectively; Figure 2D,E). Univariate Cox regression analysis revealed high *UBQLN4* mRNA expression significantly contributed to worse prognosis in RFS (hazard ratio [HR] = 2.27; 95% confidence interval [CI] 1.09–4.75; *p* = 0.029; Figure 2D) and OS (HR = 6.17; 95%CI: 1.36–28.02; *p* = 0.019; Figure 2E). This association remained evident when the analysis was restricted to patients who did not receive NAC (log rank test [*n* = 50]; *p* = 0.052 and *p* = 0.016, respectively; Figure S1A,G). In contrast, no significant differences in RFS or OS were observed when patients were stratified by conventional clinicopathological factors, including pathological stage, or lymph node metastasis (LNM), vascular invasion status, pathological differentiation (poorly vs. well + moderate), and tumor size (>5 cm vs. <5 cm) (Figure S1B–F,H–L).

In multivariable Cox proportional hazards regression analysis, high *UBQLN4* mRNA expression remained associated with shorter RFS after adjustment for LNM, vascular invasion, pathological differentiation, and tumor size (HR = 2.44; 95% CI 1.14–5.20; *p* = 0.022; Figure 2F; Table S5). To assess whether this association was dependent on median-based dichotomization, we additionally analyzed *UBQLN4* mRNA expression as a continuous variable standardized to one SD. Higher *UBQLN4* expression remained significantly associated with shorter RFS in both univariable (HR per 1-SD increase = 1.38; 95% CI 1.06–1.80; *p* = 0.016) and multivariable analyses (HR = 1.55; 95% CI 1.15–2.08; *p* = 0.004; Table S5). For OS, the direction of association was similar but did not reach statistical significance in either univariable (HR = 1.30; 95% CI 0.88–1.91; *p* = 0.187) or multivariable analysis (HR = 1.53; 95% CI 0.96–2.42; *p* = 0.072; Table S5).

### 3.3. High UBQLN4 Protein Expression Is Associated with Pathological Response to NAC in iCCA

To examine the relationship between UBQLN4 and pathological response to cisplatin-containing chemotherapy, we evaluated the protein levels of UBQLN4 in tissue samples from 16 resected-iCCA patients who received NAC (gemcitabine + cisplatin) using a multiplex immunofluorescence Ab assay (anti-CK19 and anti-UBQLN4; Table S3). Of these patients, 4 were classified as pathological responders, and 12 as non-responders. The clinicopathological characteristics and clinical outcomes of the NAC-treated patients according to pathological response status are summarized in Table S6.

**Figure 3:**
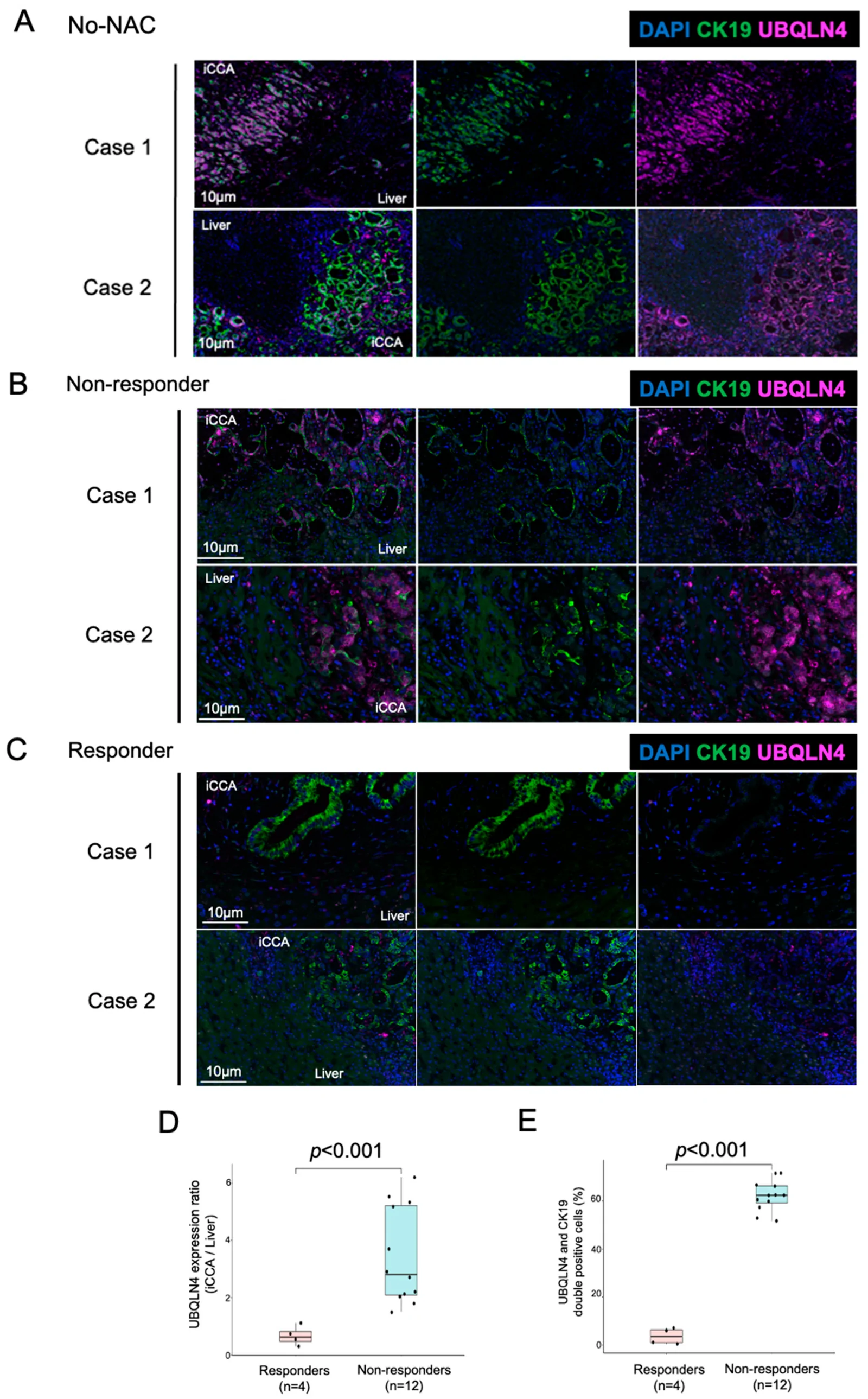
**UBQLN4 protein expression in iCCA patients can reflect NAC sensitivity.** (**A**–**C**) Representative immunofluorescence images of UBQLN4 (magenta), CK19 (green), and DAPI (blue) staining in iCCA and adjacent liver tissues from no-NAC groups (**A**), non-responders (**B**) and responders (**C**). Scale bars, 10 μm. (**D**,**E**) Quantification of UBQLN4 expression ratio (tumor vs. liver) (**D**) and percentage of UBQLN4-positive CK19+ cells (**E**), showing UBQLN4 levels in non-responders and responders. Data are presented as mean ± SD with individual data points overlaid. Statistical significance was assessed using a two-sided Welch’s unpaired *t*-test.

The protein levels of UBQLN4 were higher in iCCA tissues than in the adjacent normal liver tissues in the no-NAC groups and non-responder cases; however, UBQLN4 protein levels were lower in iCCA tumor tissues with responders compared to no-NAC groups and non-responders (Figure 3A–C). UBQLN4 protein levels ratios (iCCA tumor/adjacent normal liver tissue) were significantly higher in the non-responders (*n* = 12) than the responders’ group (*n* = 4; *p* < 0.01; Figure 3D). Additionally, the number of UBQLN4^+^ in CK19^+^ iCCA cells was significantly higher in non-responders (*n* = 12) than in responders (*n* = 4; *p* < 0.001; Figure 3E). These findings indicate that higher UBQLN4 expression was associated with poorer pathological response to NAC in this exploratory cohort.

### 3.4. UBQLN4 Overexpression Are Associated with Cell Cycle Activation in iCCA

**Figure 4:**
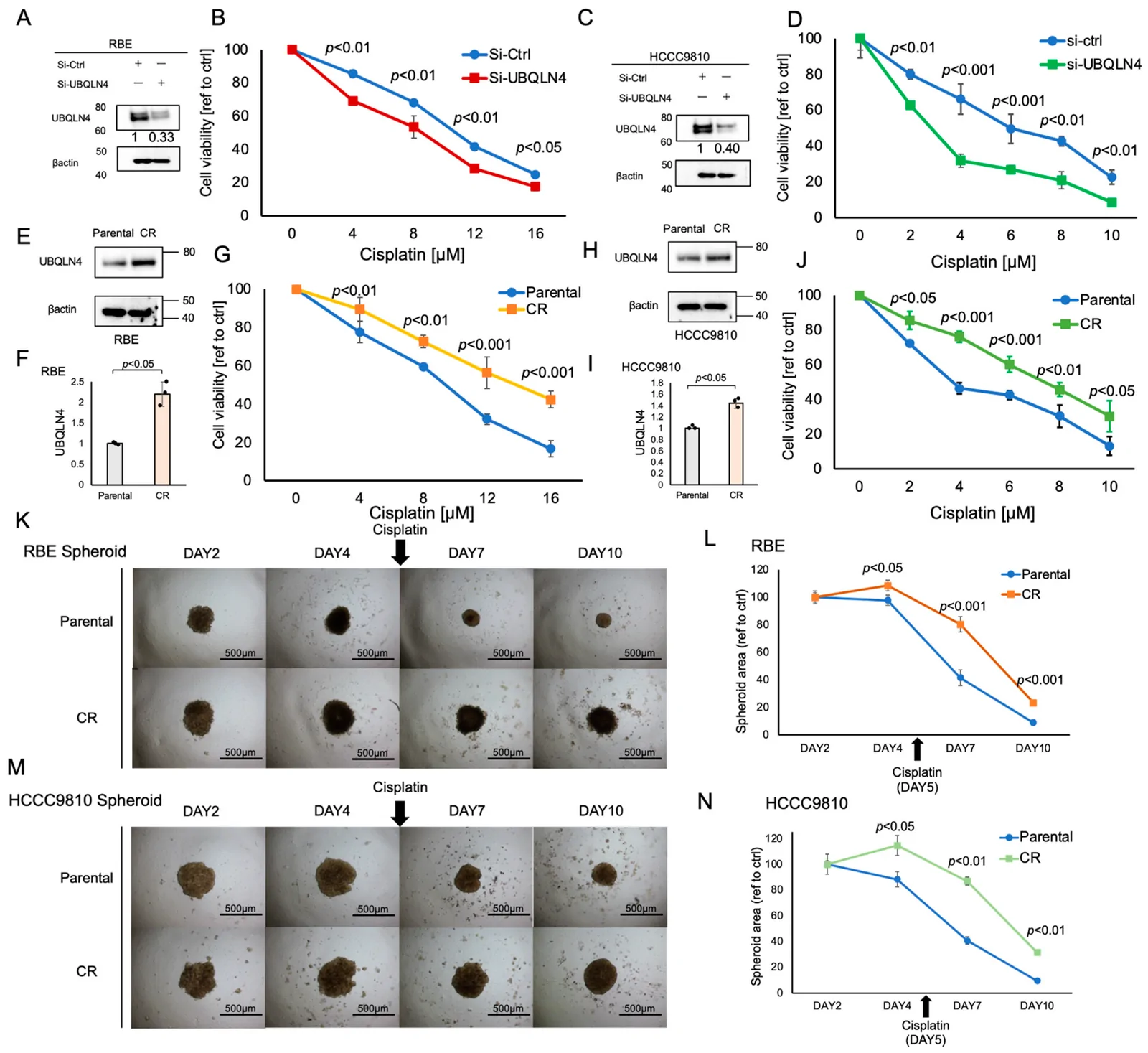
**UBQLN4 promotes cisplatin resistance in iCCA cell lines and spheroid models.** (**A**,**C**) WB analysis confirming efficient KD of *UBQLN4* in RBE (**A**) and HCCC9810 (**C**) cells. β-actin was used as a loading control. (**B**,**D**) Cell viability assays showing that *UBQLN4* KD significantly sensitizes RBE (**B**) and HCCC9810 (**D**) cells treated with si-Ctrl or si-*UBQLN4* and then exposed to cisplatin treatment with different respective concentrations. (**E**,**H**) WB analysis demonstrating UBQLN4 levels in RBE (**E**) and HCCC9810 (**H**) between CR and parental cell lines. (**F**,**I**) Quantification of UBQLN4 expression levels in parental and CR cells. (**G**,**J**) Cell viability assays in CR-RBE (**G**) and CR-HCCC9810 (**J**) cell lines treated with cisplatin. (**K**,**M**) Representative bright-field images of 3D spheroids derived from parental and CR cells treated with cisplatin (the arrow indicates treatment initiation at day 5). Spheroid growth and morphology were monitored over time. Scale bars, 500 μm. (**L**,**N**) Quantification showing the spheroid area relative to day 1 in spheroids from the respective parental and CR-iCCA cell lines following cisplatin treatment. Data are presented as mean ± SD from at least three independent experiments. Statistical significance was determined using Student’s *t*-test (**F**,**I**) or two-way ANOVA in the cell viability assay and spheroid area comparison (**B**,**D**,**G**,**J**,**L**,**N**). CR, cisplatin-resistant; KD, knockdown; WB, Western blot.

In previous studies, our group characterized the role of UBQLN4 in promoting resistance to platinum-based drugs [14,15,19]. Therefore, *UBQLN4* was KD in two iCCA cell lines (RBE and HCCC9810) to evaluate the effect on cisplatin response. Quantitative analysis of the dose–response curves further showed that UBQLN4 knockdown reduced the estimated cisplatin IC50 from 11.20 to 8.32 μM in RBE cells and from 5.44 to 2.56 μM in HCCC9810 cells, consistent with increased cisplatin sensitivity following UBQLN4 depletion. Both RBE and HCCC9810 cells with *UBQLN4*-KD showed significantly higher sensitivity to cisplatin than the respective control (Figure 4A–D), suggesting that *UBQLN4* KD may therefore shift the balance of DNA damage repair capacity, thereby sensitizing iCCA cells to cisplatin-induced genotoxic stress. Then, we investigated the association between UBQLN4 and cisplatin resistance using CR-iCCA cell lines. CR-RBE and CR-HCCC9810 were established as described in Materials and Methods. Western blot analysis revealed that both CR-RBE and CR-HCCC9810 cells expressed significantly higher UBQLN4 protein levels (Figure 4E,F,H,I). Cell viability assays demonstrated that CR-RBE and CR-HCCC9810 cells exhibited significantly higher viability than their respective parental counterparts across a wide range of cisplatin concentrations (Figure 4G,J). Quantitative analysis of the dose–response curves showed that the estimated cisplatin IC50 increased from 8.77 μM in parental RBE cells to 13.07 μM in CR-RBE cells and from 3.75 μM in parental HCCC9810 cells to 6.68 μM in CR-HCCC9810 cells, corresponding to resistance indices of 1.49 and 1.78, respectively. Then, the CR-iCCA cell lines were cultured in 3D conditions and exposed to cisplatin. As expected, spheroids derived from CR-iCCA cell lines maintained significantly larger spheroid areas compared with spheroids derived from parental cell lines, indicating increased resistance in the CR groups (Figure 4K–N). These findings indicated that CR-iCCA cell lines retain enhanced survival capacity within a 3D architecture that more closely recapitulates the in vivo tumors, supporting a stable and intrinsic resistant phenotype rather than a transient adaptive response.

## 4. Discussion

In the present study, we demonstrated that iCCA frequently harbors CN amplification of *UBQLN4* located at chromosome region 1q22, accompanied by elevated mRNA and protein expression, which is significantly associated with shorter time to postoperative recurrence, unfavorable prognosis, and decrease response to NAC regimens including cisplatin. This is an important finding in a rare type of aggressive cancer resistance to therapeutic interventions. Despite advances in genomic profiling, iCCA currently lacks robust and clinically validated biomarkers that reliably predict prognosis or therapeutic response [29], whereby existing staging systems often fail to efficiently stratify patients according to potential predicted clinical outcomes [30]. In this context, our findings showed that UBQLN4 is a molecular factor linked to unresponsive tumors and cisplatin resistance. Collectively, these results provide novel molecular insights that may help bridge the gap between genomic/transcriptomic alterations and clinically actionable treatment strategies for iCCA tumors.

Notably, the frequency of *UBQLN4* amplification identified in our cohort significantly exceeds the values previously reported and analyzing the same genetic alteration in iCCA, suggesting that *UBQLN4* amplification represents a relatively significant genomic event that relates to mRNA expression with biological relevance in iCCA tumor progression. Beyond its prevalence, UBQLN4 is functionally implicated in cisplatin resistance, highlighting its potential role in modulating tumor sensitivity to DNA-damaging agents in iCCA as well as other types of cancers [15,16,31]. UBQLN4 status may serve as a predictive marker for chemotherapy treatment responses in iCCA, particularly in NAC, which could improve triaging patients for adjuvant therapies as we demonstrated in a pilot study [19]. Further studies including larger, well-characterized patient cohorts are warranted to validate these findings and clarify the clinical significance of *UBQLN4* amplification in iCCA.

An important aspect of our findings is the significant concordance between *UBQLN4* CN amplification and its expression at both the mRNA and protein levels. Although increased gene expression in cancer cells can arise through multiple transcriptional and epigenetic mechanisms, the association observed in our study suggested genomic amplification in one key mechanism driving *UBQLN4* overexpression in iCCA. The clinical relevance of CN alterations in BTC is exemplified by *ERBB2 (HER2)* amplification, which, although detected in only a small subset of BTCs and generally in less than 5% of iCCA, has emerged as an actionable genomic alteration with the development of HER2-directed therapies [4,32,33]. Additionally, amplification of other genes, including *MET* (frequency; 1.5%) and *MDM2* (frequency; 6.5%), has been identified in subsets of BTCs and is being explored as a potential therapeutic target, although the clinical evidence remains limited [34]. Notably, the frequency of *UBQLN4* amplification observed in the TCGA-CHOL cohort was substantially higher than the reported frequency of the above gene amplifications in iCCA. Although UBQLN4 is not currently an established therapeutic target, its relatively high prevalence, association with increased expression, and relationship with clinical outcomes and chemotherapy response suggest that *UBQLN4* amplification may define a clinically relevant molecular subset of iCCA. These findings highlight the importance of further investigating recurrent CN alterations beyond currently established actionable genomic targets in iCCA.

Our findings may also have implications for patient selection for perioperative chemotherapy. The role of NAC in iCCA continues to evolve, and substantial heterogeneity exists in treatment response [35,36]. In this setting, biomarkers capable of identifying tumors unlikely to respond to platinum-based chemotherapy could be clinically valuable. Although gemcitabine plus cisplatin has long represented the backbone of systemic therapy for advanced BTC, its survival benefit remains limited, with median overall survival of approximately 11 months reported with this regimen in contemporary phase III trials [37]. More recently, the addition of immune checkpoint inhibitors, including durvalumab and pembrolizumab, to gemcitabine plus cisplatin has improved survival and established new first-line treatment options for advanced BTC [8]. Our observations indicated that patients with UBQLN4-high iCCA may derive limited benefit from conventional cisplatin-based regimens and could potentially be considered for alternative therapeutic strategies. Identifying such patients before treatment could be clinically meaningful by avoiding ineffective chemotherapy and its associated toxicity, while preventing delays in potentially more effective therapeutic approaches. Conversely, patients with low UBQLN4 expression may represent a population more likely to benefit from platinum-based treatment. Thus, UBQLN4 may have potential as a biomarker for treatment stratification as therapeutic options for iCCA continue to expand. However, these possibilities require prospective validation in larger cohorts before UBQLN4 can be incorporated into clinical decision-making.

Cancer cells are continuously exposed to proteotoxic and genotoxic stress, which may be further enhanced by cytotoxic chemotherapeutics. Increased UBQLN4 expression may therefore enhance the capacity of tumor cells to tolerate chemotherapy-induced cellular stress and promote survival under treatment. Consistent with this concept, UBQLN4 expression levels have been implicated in tumor progression and treatment resistance in several malignancies, including hepatocellular carcinoma [38], colorectal cancer [39], and non-small cell lung cancer [40], although the underlying mechanisms appear to be context-dependent. Together with our findings, these observations raise the possibility that UBQLN4 contributes to a stress-tolerant phenotype that promotes both aggressive tumor progression and resistance to cytotoxic therapy in iCCA.

Several limitations of this study should be acknowledged. First, the clinical analyses were based on a retrospective cohort with a relatively limited sample size, particularly for patients receiving NAC, and therefore require validation in larger, independent cohorts. The limited number of survival events, particularly death events, also restricts the robustness of multivariable analyses. Although UBQLN4 mRNA expression was associated with unfavorable prognosis and pathological response to cisplatin-containing chemotherapy, it remains unclear whether the latter association reflects a specific relationship with platinum sensitivity or a broader association with tumor biology and response to DNA-damaging therapy. Prospective studies with well-annotated treatment data will therefore be required to determine whether UBQLN4 has predictive value for platinum-based chemotherapy beyond its prognostic association.

Second, the functional experiments were performed in a limited number of iCCA cell lines, and UBQLN4 KD was conducted using a single targeting siRNA without rescue experiments. Thus, potential off-target effects cannot be completely excluded, and additional studies using independent genetic approaches are required to establish a causal relationship between UBQLN4 and cisplatin sensitivity. Moreover, although the CR derivatives exhibited increased IC50 values under the tested conditions, formal drug-withdrawal stability experiments, growth-kinetic analyses, and characterization of EMT or stemness phenotypes were not performed. The lack of in vivo functional validation further limits mechanistic conclusions. Finally, the pathway enrichment observed in tumors with higher UBQLN4 mRNA expression cannot establish a UBQLN4-specific causal effect and may partly reflect a broader proliferative tumor state.

## 5. Conclusions

*UBQLN4* is frequently upregulated in iCCA and is associated with aggressive clinical behavior, early postoperative recurrence, and resistance to cisplatin. These findings support UBQLN4 as a potential biomarker for risk stratification and treatment response and provide a foundation for further investigation of its role in chemoresistance in iCCA. Independent and prospective validation will be required to establish its clinical utility.

## Data Availability

The data presented in this study are available on request from the corresponding author due to privacy and ethical restrictions.

## Supplementary Materials

The following supporting information can be downloaded at: https://www.mdpi.com/article/10.3390/cells15181697/s1, Figure S1: Association of UBQLN4 expression and clinicopathological factors with survival outcomes in iCCA. (A–F) Kaplan–Meier analyses of RFS stratified by UBQLN4 expression in the No-NAC group (A), tumor stage (B), LNM status (C), vascular invasion (D), differentiation (E), and tumor size (F). (G–L) Kaplan–Meier analyses of OS according to UBQLN4 expression in the No-NAC group (G), tumor stage (H), LNM status (I), vascular invasion (J), differentiation (K), and tumor size (L). Statistical significance was determined using the log-rank test; Figure S2: Uncropped Western blot images which were used in Figure 4A,C (A–D) and Figure 4E,H (E–H); Table S1: Clinical characteristics of iCCA patients in Keio University Hospital/SJCI cohort (*n* = 66); Table S2: Key resources used in this study; Table S3: List of antibodies and dilutions utilized for multiple immunofluorescences using Opal Manual Kit; Table S4: Hallmark gene set enrichment analysis in patients with high-UBLQN4 vs. low-UBQLN4 levels from the TCGA-CHOL and GSE107943 cohorts; Table S5: Univariable and multivariable Cox proportional hazards analyses of survival outcomes in patients with iCCA; Table S6. Clinicopathological characteristics and clinical outcomes of iCCA patients receiving NAC according to pathological response.

## Abbreviations

The following abbreviations are used in this manuscript:

ANOVA	Analysis of variance
BTC	Biliary tract cancer
CI	Confidence interval
CN	Copy number
CR	Cisplatin-resistant
DDR	DNA damage response
ES	Enrichment score
ESCC	Esophageal squamous cell carcinoma
FGFR2	Fibroblast growth factor receptor 2
GISTIC	Genomic Identification of Significant Targets in Cancer
HR	Hazard ratio
iCCA	Intrahepatic cholangiocarcinoma
IC50	Half-maximal inhibitory concentration
IDH1	Isocitrate dehydrogenase 1
KD	Knockdown
LNM	Lymph node metastasis
NAC	Neoadjuvant chemotherapy
NES	Normalized enrichment score
OS	Overall survival
RFS	Relapse-free survival
TCGA	The Cancer Genome Atlas
TNBC	Triple-negative breast cancer
UBQLN4	Ubiquilin-4
WB	Western blot
