# Supplementary material for: UBQLN4 Is a Prognostic Biomarker in Intrahepatic Cholangiocarcinoma That Is Associated with Cisplatin Resistance
Source: Cells. 2026 Sep 18;15(18):1697. doi: 10.3390/cells15181697 (PMC13605264; doi:10.3390/cells15181697)
Supplement: Supplementary file 1 [file cells-15-01697-s001.zip › cells-4552592-supplementary.pdf]

Supplementary information

UBQLN4 is a prognostic biomarker in intrahepatic cholangiocarcinoma that is associated with cisplatin resistance

Short title: Clinical relevance of *UBQLN4* in iCCA

Kodai Abe[1,2], Kelly Chong,[1] Yuta Abe[2], Minoru Kitago[2], Motonori Edanami[2], Yosuke Uematsu[2], Yohei Masugi[3,4], Akihisa Ueno[4,5], Matias A. Bustos[1,6], Anton J. Bilchik[7], Yosef Shiloh[8], Yuko Kitagawa[2], Dave S. B. Hoon[1,9]

Affiliations

[1]Department of Translational Molecular Medicine, Saint John's Cancer Institute (SJCI) at Saint John's Health Center (SJHC), Providence Health System (PHS), Santa Monica, CA, 90404, USA.

[2]Department of Surgery, Keio University School of Medicine, Shinjuku, Tokyo, 1608582, Japan

[3]Department of Pathology, Keio University School of Medicine, Shinjuku, Tokyo,

1608582, Japan

[4]Department of Pathology, Tokai University School of Medicine, Isehara, Kanagawa, 2591193, Japan

[5]Division of Diagnostic Pathology, Keio University School of Medicine, Shinjuku, Tokyo, 1608582, Japan

[6] Department of Translational and Precision Medicine, SJCI at SJHC, PHS, Santa Monica, CA, 90404, USA.

[7]Department of Gastrointestinal and Hepatobiliary Surgery, SJHC, PHS, Santa Monica, CA, 90404, USA.

[8]David and Inez Myers Laboratory for Cancer Genetics, Department of Human Genetics and Computational Medicine, Gray Faculty of Medical & Health Sciences, Tel Aviv University, Tel Aviv 69978, Israel

[9] Advanced Therapeutics and Biomedical Technologies, The Lundquist Institute of Biomedical Innovation, Harbor-UCLA Medical Center, Torrance, CA 90504.

Correspondence:

Kodai Abe, MD, PhD

2200 Santa Monica Blvd, Santa Monica, CA, 90404, USA.

Email: 08medrfcab@keio.jp, abe.kodai@gmail.com

Acknowledgments

The authors thank the staff of Department of Translational Molecular Medicine and Genome Sequencing Center at SJCI for their kind advisory and technical assistance. We are also grateful to the Fourth Laboratory of the Department of Pathology in Keio University School of Medicine and SJHC pathology department for assistance with tissue processing and staining. This study was supported by the Mihashi Memorial Foundation for International Exchange Grant-in-Aid for study abroad, awarded by “Torin-kai,” the Alumni Association of the Department of Surgery, Keio University School of Medicine.

Supplementary Figures

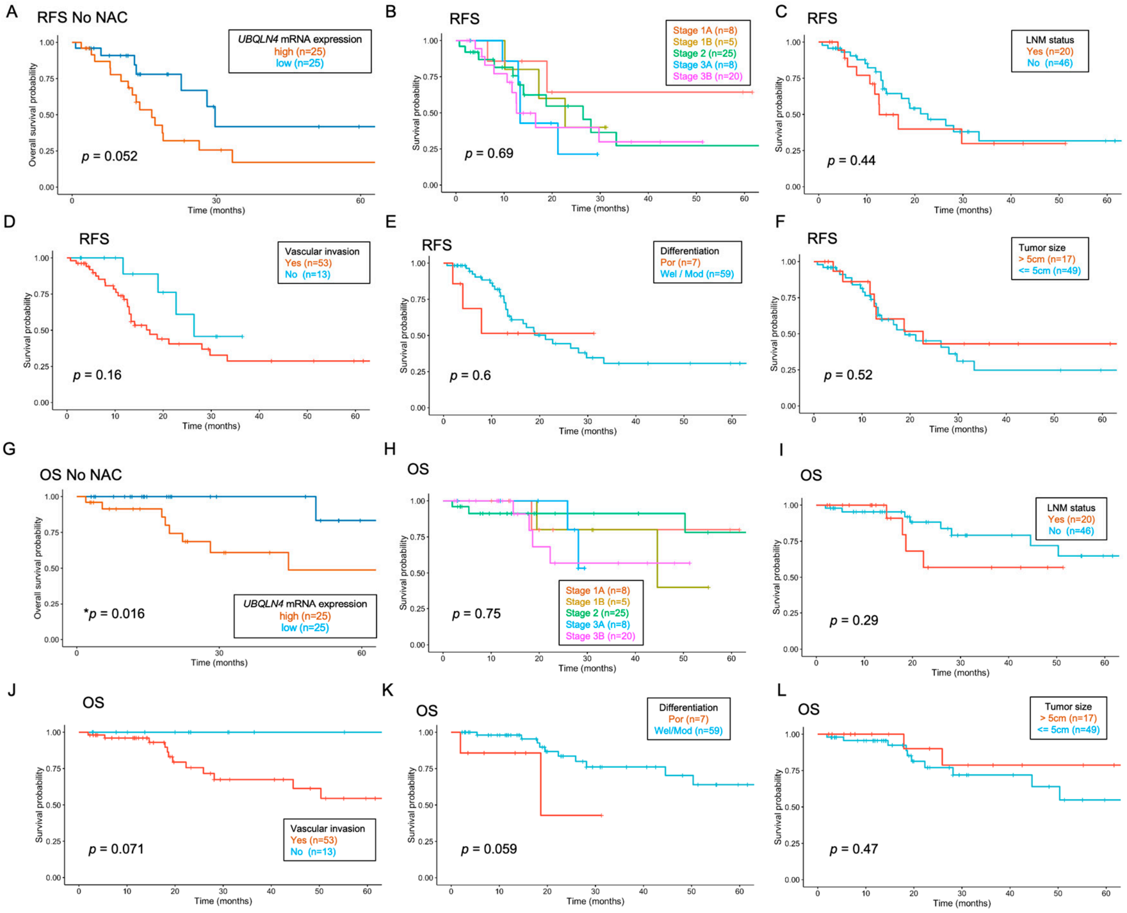

Figure S1. Association of *UBQLN4* expression and clinicopathological factors with survival outcomes in iCCA. A–F) Kaplan–Meier analyses of RFS stratified by *UBQLN4* expression in the No-NAC group **(A)**, tumor stage **(B)**, LNM status **(C)**, vascular invasion **(D)**, differentiation **(E)**, and tumor size **(F). G–L)** Kaplan–Meier analyses of OS according to *UBQLN4* expression in the No-NAC group **(G)**, tumor stage **(H)**, LNM status **(I)**, vascular invasion **(J)**, differentiation **(K)**, and tumor size **(L)**. Statistical significance was determined using the log-rank test.

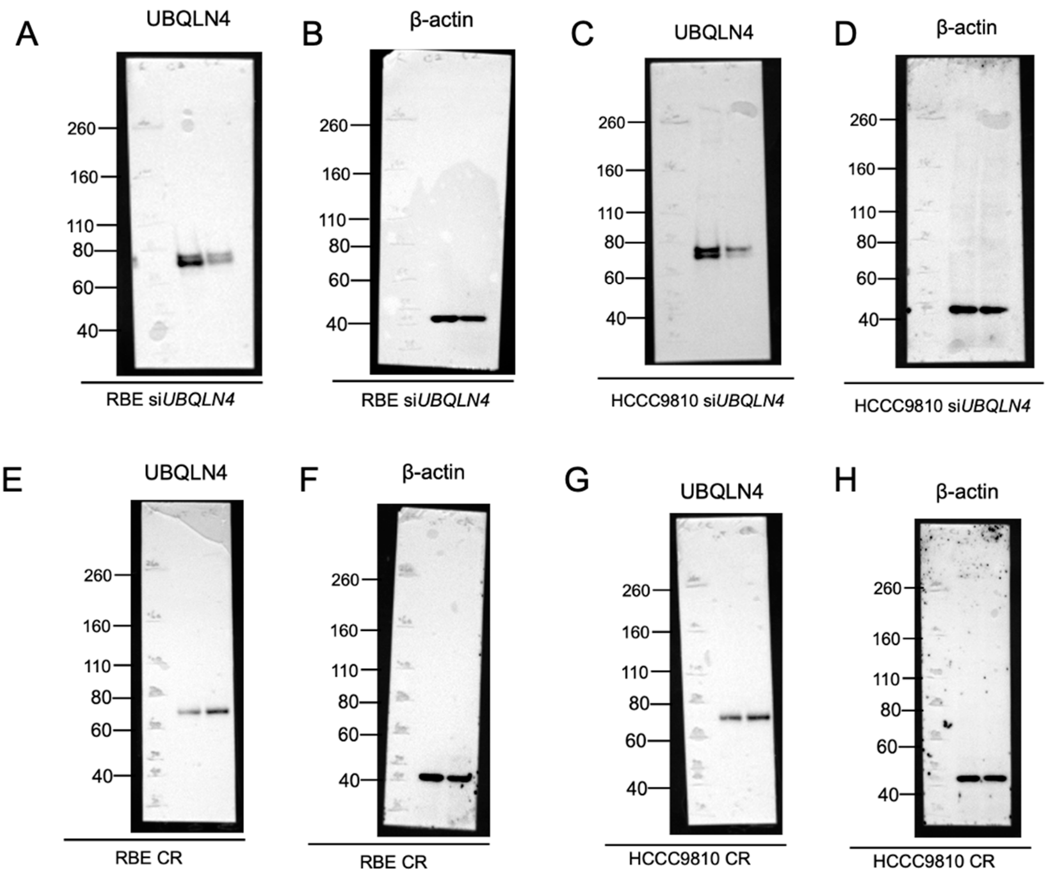

Figure. S2 Uncropped Western blot images which were used in Fig. 4A, C (A-D), Fig. 4E, H (E-H)

Table S1. Clinical characteristics of iCCA patients in Keio University Hospital/SJCI cohort (n = 66)

Characteristics	iCCA (n = 66)
Age (median, years)	69.5 (38 - 85)
Gender, n (%)	
Male	41 (62.1)
Female	25 (37.9)
Hepatitis, n (%)	
Yes	8 (12.1)
No	58 (87.9)
Neoadjuvant chemotherapy, n (%)	
Yes	16 (24.2)
No	50 (75.8)
Serum CEA (ng/mL)	2.55 (0.7-15.5)
Serum CA19-9 (U/mL)	36.0 (1.2 – 5950.55)
Surgical procedure, n (%)	
Hr0	15 (22.7)
HrS	3 (4.5)
Hr1	3 (4.5)
Hr2	31 (47.0)
Hr3	14 (21.2)
Operation time (median, min)	523 (82-1013)
Blood volume loss (median, mL)	263 (5-7150)
Tumor size (median, cm)	3.8 (1.5-12.0)
Lymph node metastasis, n (%)	
Yes	20 (30.3)
No	46 (69.7)
Vascular invasion	
Yes	53 (80.3)
No	13 (19.7)
Grade of differentiation, n (%)	
Well	7 (10.6)
Moderate	52 (78.8)
Poor	7 (10.6)
Pathological stage, n (%)	

Ia	8 (12.1)
Ib	5 (7.6)
II	25 (37.9)
IIIa	8 (12.1)
IIIb	20 (30.3)
Recurrence	
Yes	32 (48.5)
No	34 (51.5)
OS (median, range)	18.8 (1.9 - 145.8)
RFS (median,range)	13.0 (0.7 - 145.8)
Death	
Yes	13 (19.7)
No	53 (80.3)

*Values are presented as number (%) or median (range) unless stated otherwise. iCCA, intrahepatic cholangiocarcinoma; CEA, carcinoembryonic antigen; CA19-9, carbohydrate antigen 19-9; Hr, hepatic resection; OS, overall survival; RFS, recurrence free survival.

Table S2. Key resources used in this study

REAGENT or RESOURCE	SOURCE	IDENTIFIER
Antibodies		
Mouse monoclonal anti-β-actin	Sigma-Aldrich, St Louis, MO	Cat# A5441
Mouse monoclonal anti-CK19	Agilent, Santa Clara, CA	Cat# M0888
Recombinant Rabbit monoclonal anti-UBQLN4	Bethyl laboratories, Montgomery, TX	Cat# BLR145J
Biological Samples		
iCCA patients FFPE tissues	SJCI and KEIO Pathology Departments	Pathology Department in Saint John's Health Center and Keio University Hospital
Chemicals, Peptides, and Recombinant Proteins		
Cisplatin	MedChemExpress, Monmouth Junction, NJ	#HY-17394
Opal 6-Plex Manual Detection Kit	Akoya Biosciences, Brooklyn, NY	Cat#NEL81101KT
Critical Commercial Assays		
CellTiter-Glo® Luminescent Cell Viability Assay	Promega, Madison, WI	Cat# G7572
PierceTM BCA Protein Assay Kit	ThermoFisher Scientific, Waltham, MA	Cat# 23227
ZR-Duet DNA/RNA MiniPrep kit	Zymo Research, Tustin, CA	Cat# D7001
TURBO DNA-free Kit	ThermoFisher Scientific, Waltham, MA	Cat#AM1907
PerfecTa SYBR Green FastMix Reaction Mixes	VWR International, Randor, PA	Cat#101414-262
Maxwell RSC RNA	Promega, Madison, WI	Cat# AS1440

FFPE Kit		
Experimental Models: Cell Lines		
Human RBE cell line	Cytion, Sioux Falls, SD	Cat# 305019
Human HCCC-9810 cell line	Amsbio LLC, Cambridge, MA	Cat# AMS.EP-CL-0095
Transfection Reagent		
ON-TARGETplus Human UBQLN4 siRNA	Dharmacon, Lafayette, CO	Cat# L-021178-01-0005
ON-TARGETplus Non-targeting pool siRNA	Dharmacon, Lafayette, CO	Cat# L-017599-00-0005
JetPRIME	VWR International, Randor, PA	Cat# 89129-924
Software and Algorithms		
ImageJ	NIH, Bethesda, MD	imagej.nih.gov/ij/
Adobe Illustrator	Adobe, Sannose, CA	www.adobe.com
inForm 2.6.0	Akoya Biosciences, Brooklyn, NY	www.akoyabio.com/
Mantra Snap 1.05	Perkin Elmer, Waltham, MA	www.perkinelmer.com
Primer Sequences (5'-3') for RT-qPCR	Integrated DNA Technologies, Coralville, IA	
UBQLN4	F: CAGATGCTGGCAGTGGAA	R: CCAAAGCCAGAGAGTATGGAC
SDHA	F: TCAGCATGCAGAAGTCAAT	R: GAACGTCTTCAGGTGCTTT

Table S3. List of antibodies and dilutions utilized for multiple immunofluorescences using Opal Manual Kit.

Staining order	Ab name	Class	Type	Dilution	Cat#	Supplier	Opal Fluorophore	Opal dilution
1	CK19	IgG	Mouse monoclonal	1:200	M0888	Agilent	520	1:150
2	UBQLN4	IgG	Rabbit monoclonal	1: 500	BLR145J	Bethyl laboratories	690	1:150
3	DAPI	-	-	1:1000	62248	ThermoFisher	-	-

Table S4. Hallmark gene set enrichment analysis in patients with high-*UBLQN4* vs. low-*UBQLN4* levels from the TCGA-CHOL and GSE107943 cohorts.

A. TCGA-CHOL

	Pathway	ES	NES	p-value	FDR-adjusted p-value	Size	Enrichment
1	HALLMARK_MITOTIC_SPINDLE	0.427	1.432	0.003	0.011	199	Up-regulated
2	HALLMARK_G2M_CHECKPOINT	0.403	1.352	0.010	0.030	200	Up-regulated
3	HALLMARK_MTORC1_SIGNALING	-0.390	-1.317	0.019	0.048	199	Down-regulated
4	HALLMARK_FATTY_ACID_METABOLISM	-0.426	-1.387	0.011	0.031	157	Down-regulated
5	HALLMARK_COMPLEMENT	-0.412	-1.392	0.009	0.028	200	Down-regulated
6	HALLMARK_APOPTOSIS	-0.436	-1.429	0.006	0.020	161	Down-regulated
7	HALLMARK_INFLAMMATORY_RESPONSE	-0.424	-1.433	0.004	0.015	199	Down-regulated
8	HALLMARK_PEROXISOME	-0.463	-1.437	0.012	0.033	104	Down-regulated
9	HALLMARK_XENOBIOTIC_METABOLISM	-0.431	-1.456	0.003	0.011	199	Down-regulated
10	HALLMARK_SPERMATOGENESIS	-0.466	-1.488	0.002	0.011	132	Down-regulated
11	HALLMARK_IL2_STAT5_SIGNALING	-0.441	-1.489	0.002	0.011	198	Down-regulated
12	HALLMARK_P53_PATHWAY	-0.460	-1.556	<0.001	0.003	199	Down-regulated
13	HALLMARK_ESTROGEN_RESPONSE_LATE	-0.475	-1.603	<0.001	0.001	198	Down-regulated
14	HALLMARK_DNA_REPAIR	-0.512	-1.657	<0.001	0.001	149	Down-regulated
15	HALLMARK_INTERFERON_GAMMA_RESPONSE	-0.502	-1.698	<0.001	<0.001	199	Down-regulated
16	HALLMARK_PANCREAS_BETA_CELLS	-0.641	-1.724	0.002	0.011	40	Down-regulated
17	HALLMARK_ADIPOGENESIS	-0.525	-1.772	<0.001	<0.001	200	Down-regulated
18	HALLMARK_ALLOGRAFT_REJECTION	-0.551	-1.862	<0.001	<0.001	200	Down-regulated
19	HALLMARK_OXIDATIVE_PHOSPHORYLATION	-0.568	-1.918	<0.001	<0.001	200	Down-regulated

B. GSE107943

	Pathway	ES	NES	p-value	FDR-adjusted p-value	Size	Enrichment
1	HALLMARK_MYC_TARGETS_V2	0.701	2.109	<0.001	<0.001	57	Up-regulated
2	HALLMARK_MYC_TARGETS_V1	0.523	1.883	<0.001	<0.001	193	Up-regulated
3	HALLMARK_E2F_TARGETS	0.509	1.834	<0.001	<0.001	195	Up-regulated
4	HALLMARK_DNA_REPAIR	0.491	1.714	<0.001	<0.001	147	Up-regulated
5	HALLMARK_CHOLESTEROL_HOMEOSTASIS	0.542	1.703	0.001	0.003	73	Up-regulated
6	HALLMARK_PEROXISOME	0.509	1.683	<0.001	0.001	104	Up-regulated
7	HALLMARK_UNFOLDED_PROTEIN_RESPONSE	0.444	1.473	0.005	0.011	106	Up-regulated
8	HALLMARK_REACTIVE_OXYGEN_SPECIES_PATHWAY	0.509	1.470	0.011	0.020	47	Up-regulated
9	HALLMARK_WNT_BETA_CATENIN_SIGNALING	0.512	1.449	0.027	0.046	42	Up-regulated
10	HALLMARK_G2M_CHECKPOINT	0.397	1.430	0.004	0.009	190	Up-regulated
11	HALLMARK_FATTY_ACID_METABOLISM	0.390	1.369	0.011	0.020	155	Up-regulated
12	HALLMARK_UV_RESPONSE_UP	0.369	1.294	0.026	0.046	154	Up-regulated
13	HALLMARK_TGF_BETA_SIGNALING	-0.530	-1.520	0.010	0.020	54	Down-regulated
14	HALLMARK_APICAL_JUNCTION	-0.443	-1.526	0.001	0.003	191	Down-regulated
15	HALLMARK_COMPLEMENT	-0.456	-1.575	<0.001	0.001	199	Down-regulated
16	HALLMARK_HYPOXIA	-0.480	-1.653	<0.001	<0.001	191	Down-regulated
17	HALLMARK_UV_RESPONSE_DN	-0.546	-1.800	<0.001	<0.001	138	Down-regulated
18	HALLMARK_IL2_STAT5_SIGNALING	-0.535	-1.844	<0.001	<0.001	194	Down-regulated
19	HALLMARK_ALLOGRAFT_REJECTION	-0.565	-1.953	<0.001	<0.001	195	Down-regulated
20	HALLMARK_KRAS_SIGNALING_UP	-0.571	-1.966	<0.001	<0.001	193	Down-regulated
21	HALLMARK_INTERFERON_ALPHA_RESPONSE	-0.695	-2.181	<0.001	<0.001	95	Down-regulated
22	HALLMARK_INFLAMMATORY_RESPONSE	-0.648	-2.240	<0.001	<0.001	197	Down-regulated

23	HALLMARK_TNFA_SIGNALING_VIA_NFKB	-0.656	-2.268	<0.001	<0.001	197	Down-regulated
24	HALLMARK_INTERFERON_GAMMA_RESPONSE	-0.684	-2.364	<0.001	<0.001	197	Down-regulated
25	HALLMARK_EPITHELIAL_MESENCHYMAL_TRANSITION	-0.703	-2.429	<0.001	<0.001	195	Down-regulated

ES, enrichment score; NES, normalized enrichment score; FDR, false discovery rate. Positive and negative NES values indicate enrichment in tumors with higher and lower *UBQLN4* expression, respectively. Pathways with an FDR-adjusted P value < 0.05 are shown.

Table S5. Univariable and multivariable Cox proportional hazards analyses of survival outcomes in patients with iCCA

Variable	Univariable HR (95% CI)	*p*-value	Multivariable HR (95% CI)	*p*-value
Primary analysis for RFS				
UBQLN4 mRNA levels (High vs. Low)	2.24 (1.07 - 4.68)	0.033	2.44 (1.14 - 5.20)	0.022
Lymph node metastasis (Yes vs. No)	1.36 (0.64 - 2.89)	0.432	1.56 (0.69 - 3.50)	0.285
Vascular invasion (Yes vs. No)	2.10 (0.73 - 6.02)	0.168	1.85 (0.63 - 5.40)	0.263
Differentiation (poor vs. well/moderate)	1.38 (0.42 - 4.55)	0.601	1.60 (0.47 - 5.40)	0.453
Tumor size (>5 vs. ≤5 cm)	0.75 (0.32 - 1.76)	0.511	0.71 (0.29 - 1.70)	0.455
Sensitivity analyses using continuous *UBQLN4* expression				
RFS: *UBQLN4* mRNA levels (per 1-SD increase)	1.38 (1.06–1.80)	0.016	1.55 (1.15 - 2.08)	0.004
OS: *UBQLN4* mRNA levels (per 1-SD increase)	1.30 (0.88–1.91)	0.187	1.53 (0.96–2.42)	0.072

iCCA, intrahepatic cholangiocarcinoma; HR, hazard ratio; CI, confidence interval; SD, standard deviation; RFS, recurrence-free survival; OS, overall survival. A total of 32 recurrence events and 13 death events were observed among 66 patients. *UBQLN4* mRNA expression was dichotomized at the median in the primary RFS analysis. As sensitivity analyses, *UBQLN4* mRNA expression was analyzed as a continuous variable standardized to one SD for both RFS and OS. The multivariable sensitivity analyses were adjusted for the same clinicopathological covariates as the primary RFS model. Given the limited number of death events, the multivariable OS analysis should be interpreted cautiously.

Table S6. Clinicopathological characteristics and clinical outcomes of iCCA patients receiving NAC according to pathological response

Characteristics	Non-responders (n = 12)	Responders (n = 4)
Age (median, years)	74 (51 - 82)	70.5 (62 - 81)
Clinical stage, n (%)		
IIIa	7 (58.3)	1 (25)
IIIb	5 (41.7)	3 (75)
Tumor size (median, cm)	4.3 (1.5 - 8.8)	5.1 (4.0 - 5.7)
Vascular invasion		
Yes	12	2 (50)
No	0	2 (50)
Lymph node metastasis, n (%)		
Yes	5 (41.7)	3 (75)
No	7 (58.3)	1 (25)
Grade of differentiation, n (%)		
Well/Moderate	12	2 (50)
Poor	0	2 (50)
Recurrence		
Yes	7 (58.3)	1 (25)
No	5 (41.7)	3 (75)
OS (median, months)	15.1 (2.3 - 61.7)	23.4 (14.6 - 36.5)
RFS (median, months)	10.8 (2.3 - 61.7)	23.4 (10.6 - 36.5)
Death		
Yes	2 (16.7)	1 (25)
No	10 (83.3)	3 (75)

*Values are presented as number (%) or median (range) unless stated otherwise. iCCA, intrahepatic cholangiocarcinoma; NAC, neoadjuvant chemotherapy; OS, overall survival; RFS, recurrence free survival.
